# Cortical thickness contributes to cognitive heterogeneity in patients with type 2 diabetes mellitus

**DOI:** 10.1097/MD.0000000000010858

**Published:** 2018-05-25

**Authors:** Chang Li, Chuanming Li, Qifang Yang, Bin Wang, Xuntao Yin, Zhiwei Zuo, Xiaofei Hu, Yuqi Lai, Jian Wang

**Affiliations:** a Department of Radiology, Southwest Hospital, Third Military Medical University (Army Medical University), Chongqing; b Department of Internal Medicine, The Second Outpatient Department of Chengdu Army Region Authority, Chengdu; c School of Foreign Languages and Cultures, Chongqing University, Chongqing, China.

**Keywords:** cortical thickness, FreeSurfer, mild cognitive impairment (MCI), MRI, type 2 diabetes mellitus (T2DM)

## Abstract

The aim of this study was to investigate cerebral cortical thickness alterations in patients with type 2 diabetes mellitus (T2DM) and their association with mild cognitive impairment (MCI).

Thirty T2DM patients without MCI, 30 T2DM patients with MCI, and 30 healthy controls were recruited. All subjects underwent high-resolution sagittal T1-weighted structural imaging using a 3-dimensional magnetization prepared rapid acquisition gradient echo (MPRAGE) sequence. The cortical thicknesses of the whole brain of the 3 groups were analyzed and compared using analysis of variance (ANOVA) test. Partial correlations between the cortical thicknesses of each brain region and standard laboratory testing data were analyzed for the T2DM without MCI group. The associations between cortical thicknesses and neuropsychological scale scores were also analyzed in the T2DM with MCI group.

Compared with the healthy controls, the T2DM without MCI group showed statistically significant reduction in the cortical thickness of the left posterior cingulate gyrus, right isthmus cingulate gyrus, middle temporal gyrus, paracentral lobule, and transverse temporal gyrus. No significant correlation was found between the standard laboratory testing data and the cortical thicknesses of these cerebral regions. Compared with the T2DM without MCI group, the cortical thickness alterations in the T2DM with MCI group were bidirectional. Increased cortical thickness was found in the left parahippocampal gyrus and the right isthmus cingulate gyrus. Decreased cortical thickness was observed in the left pars triangularis and the right pars opercularis. Significant correlations were found between the cortical thickness of the right pars opercularis and the Complex Figure Test-delayed recall scores (*r* = 0.464, ρ = 0.015), Trail Making Test A consuming time (*r* = −0.454, ρ = 0.017), and Montreal Cognitive Assessment scores (*r* = 0.51, ρ = 0.007).

T2DM could influence the gray matter of several brain regions. The cortical thickness reduction of the right pars opercularis may be a biomarker of cognitive impairment and play an important role in its pathophysiological mechanism.

## 1 Introduction

Type 2 diabetes mellitus (T2DM) is a common metabolic disorder characterized by insulin resistance and hyperglycemia. Insulin resistance and hyperglycemia often impair cognitive functions and significantly increase the risk of dementia.^[1,2]^ Approximately 10.8% to 17.5% of T2DM patients can develop cognitive dysfunction,^[3–5]^ from mild cognitive impairment (MCI) to dementia.^[6]^ Dementia is irreversible, which will cause a long-term decrease in the ability to learn, think, and remember and greatly affects the daily functions of the patient. MCI is the state between normal and dementia, which is reversible and attracts increasing research interest recently. Therefore, a study of the pathophysiological mechanisms of MCI related to T2DM is important for early diagnosis and timely treatment of T2DM patients.

MRI has been proven as an effective method to study the cerebrum morphology and is widely used as a research method to detect and evaluate neural diseases. Previously, the total cortical volume of both hemispheres was found consistently decreased in the T2DM patients.^[7,8]^ The atrophic cortical areas were mainly distributed in the frontal lobe including the right superior frontal gyrus and the left paracentral lobule.^[9]^ In another study, the cortical thickness has been observed thinned in the bilaterally middle temporal gyrus and posterior cingulate gyrus for patients with T2DM compared with healthy controls (HCs).^[10]^

However, previous studies mainly focused on the regional abnormalities in the cerebral cortical morphology of T2DM. In this study, we subdivided T2DM patients into T2DM with MCI and T2DM without MCI groups to explore potential cerebral cortical thickness alterations and its possible relationship with cognitive impairment, based on the fact that cortical thickness contributes to cognitive heterogeneity in patients with T2DM. We hypothesized that alterations in cortical thickness in T2DM patients could be located in multiple brain regions and individual cortical thickness alteration in particular regions could correlate with the cognitive impairment of T2DM.

## 2 Materials and methods

This study was carried out in accordance with the Declaration of Helsinki. The protocol was approved by the Medical Ethics Committee of Southwest Hospital. All subjects gave written informed consent.

### 2.1 Participants

Thirty patients with T2DM without MCI (T2DM without MCI) and 30 patients with T2DM and MCI were recruited from our hospital between October 2015 and November 2016 (Table 1). T2DM was diagnosed using the 1999 criteria proposed by the World Health Organization .^[11]^ The diagnosis of MCI was based on the criteria established in the 2006 European Alzheimer's Disease Consortium,^[12]^ which includes complaints of hypomnesis, MoCA score < 26, mini mental state exam (MMSE) score > 24, clinical dementia rating (CDR) ≥ 0.5, and normal activities of daily living (ADL) score. Participants were excluded if they had a history of brain injury, alcoholism, epilepsy, Parkinson disease, major depression, or other psychiatric or neurological disorder. Participants with dementia (MMSE ≤ 24), severe depression (Hamilton Depression Rating Scale ≥ 18), severe claustrophobia, or contraindications to magnetic resonance imaging (MRI) were also excluded. T2DM patients were excluded if they had microvascular complications, including nephropathy, retinopathy, and neuropathy.^[9,10]^ Thirty volunteers without vascular risk factors, nervous system diseases, cognitive complaints, or psychiatric illnesses were recruited as HCs. Thirty HCs had no T2DM, psychiatric or neurological disorder, and had MoCA score ≥ 26, MMSE score > 24. Height, weight, and body mass index (BMI) were measured for each participant. All participants were tested by neurological, neuropsychological and structural MRI examinations. All participants were right-handed and signed informed consent before the study started.

**Table 1:**
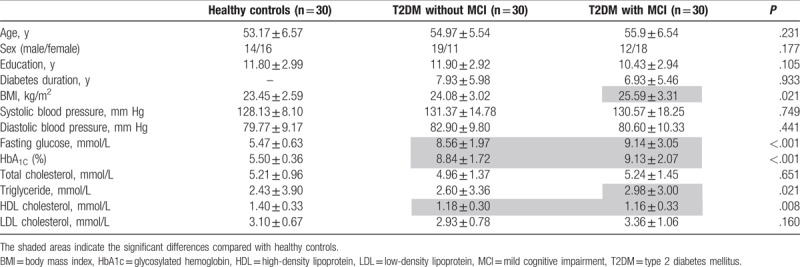
Demographic and clinical standard laboratory tests data of all subjects.

#### 2.1.1 Neuropsychological assessments

The neuropsychological assessment included the MMSE, Montreal Cognitive Assessment (MoCA), Auditory Verbal Learning Test (AVLT), Digit Symbol Coding Test (DSCT), Complex Figure Test (CFT), Trail-Making Test (TMT), Digit Span Test (DST), and Verbal Fluency Test (VFT).

#### 2.1.2 Standard laboratory tests

Standard laboratory tests were performed to evaluate glycosylated hemoglobin (HbA1c), fasting plasma glucose (FPG), fasting C-peptide, fasting insulin, triglycerides (TGs), total cholesterol (TC), low-density lipoprotein (LDL), high-density lipoprotein (HDL), homocysteine, urinary microalbumin, blood urea nitrogen (BUN), uric acid, serum creatine, cystatin C, free thyroxine (FT), free triiodothyronine (FT3), and thyroid-stimulating hormone (TSH).

### 2.2 MR image acquisition

All imaging data were obtained on a 3-Tesla Trio MRI system (Siemens Healthcare, Erlangen, Germany) equipped with a 12-channel phase-array head coil. The subjects were requested to keep calm and avoid any movement during the image acquisition with their eyes closed. The 3D high-resolution structural images were obtained using a T1-weighted magnetization prepared rapid acquisition gradient echo (MPRAGE) sequence (repetition time = 1900 ms, echo time = 2.52 ms, inversion time = 900 ms, flip angle = 9°, matrix = 256 × 256, thickness = 1.0 mm, 176 slices with voxel size = 1 mm x 1 mm x 1 mm). Then, all the subjects were required to undergo conventional brain T1-weighted and fluid-attenuated inversion recovery (FLAIR) images to exclude organic diseases and white matter (WM) hyperintense lesions. The T1-weighted images were obtained using the following parameters: TR/TE = 200/2.78 ms, flip angle = 70°, matrix = 384 × 384, thickness = 4.0 mm, 25 slices, voxel size = 0.7 × 0.6 × 5 mm^3^. The FLAIR sequence was scanned using the following parameters: TR/TE/TI = 9000/93/2500 ms, flip angle = 130°, matrix = 256 × 256, thickness = 4.0 mm, 25 slices, voxel size = 0.9 × 0.9 × 4 mm^3^.

### 2.3 Image processing

The data were exported from the MRI scanner to a personal computer on which off-line analysis was performed and operating system is Linux Operating System. Before further analysis of the 3D brain images, we first confirmed that all images were not affected by head motion. Then, all data were converted to MGZ (compressed Massachusetts General Hospital file) format, and the whole brain cortical thickness was measured at each point of the cortical mantle using FreeSurfer software (version 5.3.0, available at http://surfer.nmr.mgh.harvard.edu). The automated processing stream of FreeSurfer had multiple steps, including Talairach transformation, removal of nonbrain tissue, automatic correction of topological defects, inflation of the folded surface, and registration into an average spherical surface template. A deformable surface algorithm was used to segment the gray/WM tissue and cerebrospinal fluid (CSF) with submillimeter precision. The cortical areas were segmented into 34 regions in each hemisphere.^[13]^ The average of the shortest distances between the 2 boundaries of each region equaled the cortical thickness.

### 2.4 Statistical analyses

Statistical analyses were performed using Statistical Product and Service Solutions software (version 18.0; SPSS, Chicago, IL). If the data distribution was normal, comparisons among the 3 groups were performed by using the analysis of variance (ANOVA) test and the results were corrected by LSD (Least Significant Difference). If the data distribution was not normal, the Kruskal–Wallis *H* test and Mann–Whitney *U* test were employed for nonparametric analysis. A *P* value of .05 was considered statistically significant. Partial correlations between the cortical thickness of each brain region and standard laboratory testing data were analyzed for the T2DM without MCI group and the T2DM with MCI group. Partial correlations between the cortical thickness of each brain region and the neuropsychological scale scores were analyzed for the T2DM with MCI group. Age, gender, and education level of each subject were imported as covariates. The demographic and standard laboratory tests include age, gender, education, BMI, systolic blood pressure, diastolic blood pressure, fasting glucose, HbA1C, TC, TG, HDL cholesterol, LDL cholesterol; the Neuropsychological scores include the MMSE, MoCA, AVLT, DSCT, CFT, TMT, DST, VFT, and the cortical thickness of each brain region was compared among the 3 groups.

## 3 Results

### 3.1 Demographic and standard laboratory test differences

Compared with the HCs, the T2DM without MCI group had higher FPG and HbA1c and lower HDL scores. The T2DM with MCI group had higher BMI, FPG, HbA1c, TG, and lower HDL scores. There were no significant differences in standard laboratory tests between T2DM without MCI group and T2DM with MCI group. No significant differences were found in age, gender, education, systolic or diastolic blood pressure, LDL, TC among the 3 groups (Table 1).

### 3.2 Neuropsychological differences

**Figure 1:**
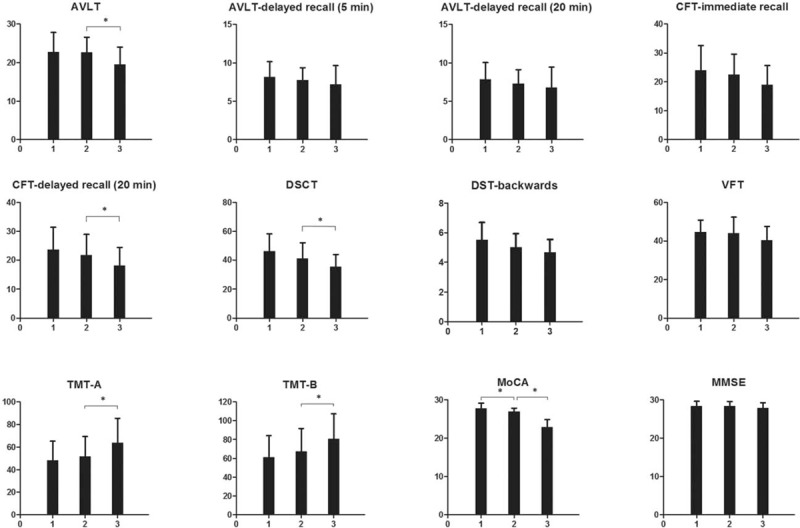
Neuropsychological scale scores among the 3 groups. 1 is HC group; 2 is T2DM without mild cognitive impairment group; 3 is T2DM with mild cognitive impairment group. The Y-axes in the tables of TMT-A and TMT-B indicate the time (second) the participants spent in completing the tests. The Y-axes in the other tables indicate the neuropsychological scale scores. ^∗^*P* < .05.

Compared with the HCs, the T2DM without MCI group had lower MoCA scores. The T2DM with MCI group had higher TMT-A consuming time, TMT-B consuming time, and lower AVLT scores, CFT-immediate recall scores, CFT-delayed recall scores, DSCT scores, DST-backwards scores, and MoCA scores. Compared with the T2DM without MCI group, the T2DM with MCI group had higher TMT-A consuming time, TMT-B consuming time and lower AVLT scores, CFT-delayed recall (20 minutes) scores, DSCT scores, and MoCA scores. There were no significant differences in AVLT-delayed recall (5 minutes) scores, AVLT-delayed recall (20 minutes) scores, VFT scores, or MMSE scores (*P* *>* .05) among the 3 groups (Fig. 1).

### 3.3 Cortical thickness differences

#### 3.3.1 Healthy controls versus T2DM without MCI

**Figure 2:**
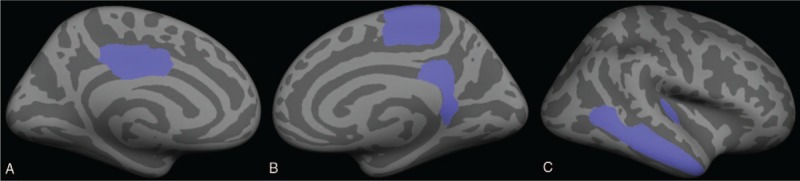
Pattern of cortical thickness alterations in T2DM without MCI patients compared with the HC group. (A) Medial view of left hemisphere; (B) medial view of right hemisphere; and (C) lateral view of right hemisphere. Blue colors indicate areas with cortical thickness reductions.

Compared with the HC group, the T2DM without MCI group showed statistically significant cortical thickness reductions in the left posterior cingulate gyrus, right isthmus cingulate gyrus, middle temporal gyrus, paracentral lobule, and transverse temporal gyrus (Table 2, Fig. 2). No correlation was found between the standard laboratory testing data and the cortical thickness of these cerebral regions.

**Table 2:**
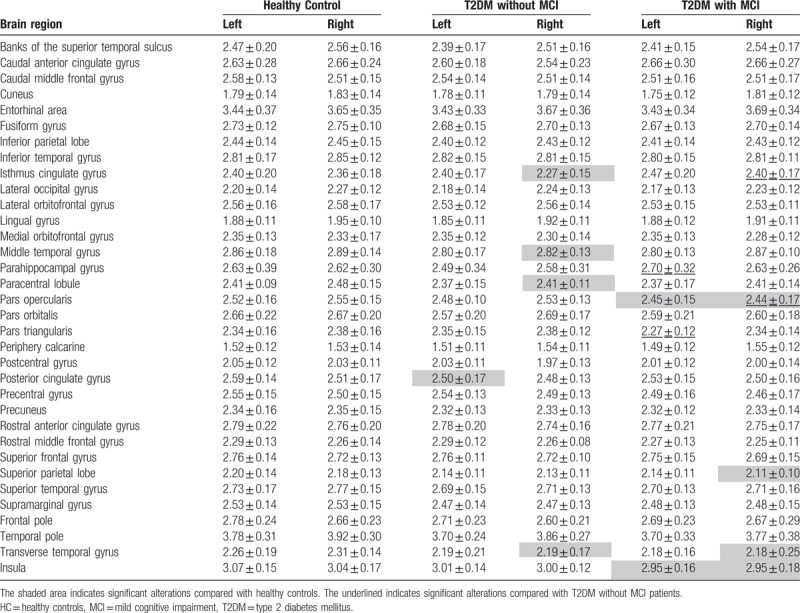
Cortical thickness in HC, T2DM without MCI, and T2DM with MCI groups (mean ± standard deviation, mm).

### 3.4 T2DM without MCI versus T2DM with MCI

**Figure 3:**
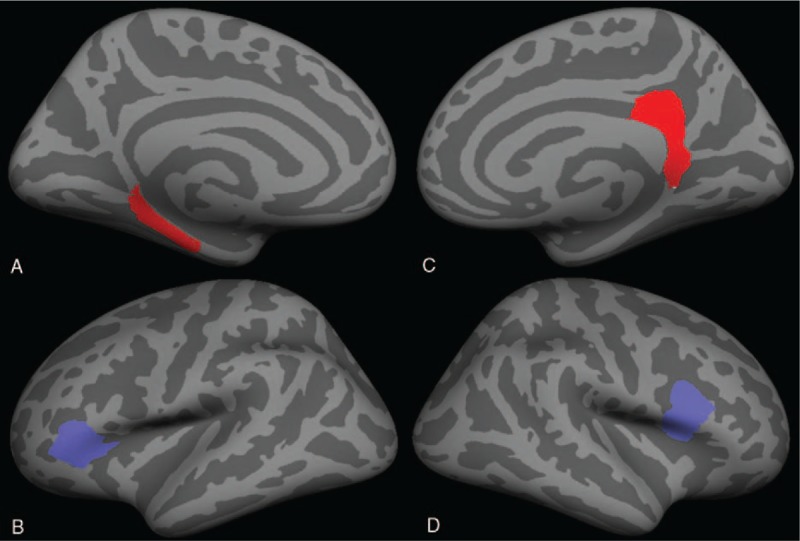
Pattern of cortical thickness alterations in T2DM with MCI patients compared with T2DM without MCI patients. (A) Medial view of left hemisphere; (B) lateral view of left hemisphere; (C) medial view of right hemisphere; and (D) lateral view of right hemisphere. Blue and red colors indicate areas with cortical thickness reduction or increase, respectively.

**Figure 4:**
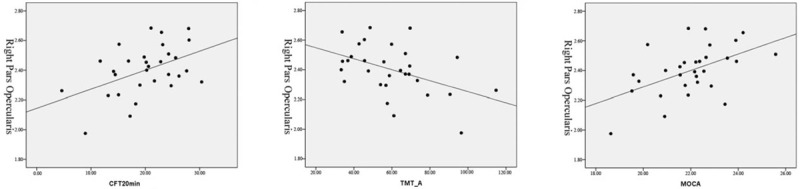
Correlations between the cortical thickness of the right pars opercularis and the CFT-delayed recall (20 min) scores, TMT-A consuming time, and MoCA scores in the T2DM with MCI patients. The partial correlation coefficient (ρ) was corrected for age, gender, and education.

Compared with the T2DM without MCI group, the cortical thickness alterations of the T2DM with MCI group were bidirectional. Cortical thickness increasing was found in the left parahippocampal gyrus and right isthmus cingulate gyrus. Cortical thickness reduction was observed in the left pars triangularis and right pars opercularis (Table 2, Fig. 3). A close relationship was found between the cortical thickness of the right pars opercularis and the CFT-delayed recall (20 minutes) scores (*r* = 0.464, ρ = 0.015), TMT-A consuming time (*r* = -0.454, ρ = 0.017), and MoCA scores (*r* = 0.51, ρ = 0.007) of the T2DM with MCI group (Fig. 4).

### 3.5 Healthy controls versus T2DM with MCI

Compared with the HC group, the T2DM with MCI group showed statistically significant cortical thickness reduction in the bilateral pars opercularis, bilateral insula, right superior parietal lobule, and right transverse temporal gyrus. No correlation was found between the standard laboratory testing data and the cortical thickness of these cerebral regions in T2DM with MCI.

## 4 Discussion

Our study is to investigate cerebral cortical thickness alterations related to T2DM-related MCI using FreeSurfer. The research disclosed several findings. Compared with the T2DM without MCI group, the cortical thickness alterations of the T2DM with MCI group were bidirectional. Decreased cortical thickness was observed in the left pars triangularis and the right pars opercularis. The pars opercularis has many important functions in the brain. It is involved in semantic processing, language production, and phonological processing. Both the pars opercularis and the pars triangularis are involved in higher cognitive functions such as memory,^[14]^ emotion, and learning.^[15]^ The cortical thickness of the right pars opercularis was closely correlated with several cognitive tests scores. Our results suggest that cortical thickness reduction of the right pars opercularis may be a biomarker of cognitive impairment and play an important role in the pathophysiological mechanism. Measuring the cortical thickness of the right pars opercularis may help detect and evaluate the cognitive impairment of T2DM patients.

Compared with the T2DM without MCI group, increased cortical thickness in the left parahippocampal gyrus and right isthmus cingulate gyrus was also found in the T2DM with MCI patients. The increased cortical thickness of these cerebral regions may represent a compensation for the cognitive decline.^[16,17]^ The left parahippocampal gyrus and right isthmus cingulate gyrus both play important roles in cognition. The parahippocampal gyrus is responsible for memory encoding and recognition.^[18]^ The isthmus cingulate gyrus has been proven to be involved in emotion formation, learning, and memory.^[19]^

Compared with HCs, the T2DM without MCI patients showed significantly reduced cortical thickness in the left posterior cingulate gyrus, right isthmus cingulate gyrus, middle temporal gyrus, paracentral lobule, and transverse temporal gyrus. However, no close correlation was found between all laboratory testing data and the cortical thickness of these cerebral regions. Our results suggest that brain morphology of the above area may be affected by T2DM. However, the brain morphology alterations are not parallel with the clinical manifestations. The cingulate gyrus is responsible for human awareness, episodic memory retrieval, emotion processing, learning, and memory.^[19,20]^ The middle temporal gyrus connects with the recognition of known faces, accessing the meaning of words while reading and social problem solving.^[21,22]^ The paracentral lobule is involved in the sensory and motor innervations of the contralateral lower extremity. The transverse temporal gyrus plays important roles in auditory processing. Our results are generally consistent with previously published literature.^[10,23,24]^ Lastly, cortical thickness reductions have also been observed in the middle temporal gyrus, posterior cingulate gyrus in T2DM patients.^[10]^ Peng et al^[9]^ also reported cortical thickness reductions in the temporal gyrus in T2DM patients. Reductions in the cingulate region in T2DM subjects have also been found in the literature.^[14]^ T2DM may affect cortical morphology through several different mechanisms. Chronic hyperglycemia, repeated hypoglycemic episodes, vascular disease, and possibly the direct effects of insulin on the brain may all be implicated.^[25–27]^ Vascular impairment induced by diabetes mellitus could play an important role in the etiology of brain atrophy.^[28–30]^ Blood glucose levels were also proven to be associated with cortical thinning.^[31,32]^ The dysregulation of glycemic variability caused by T2DM may also contribute to brain atrophy.^[33]^

Some limitations of our study should be noted. First, this is a cross-sectional clinical research with a relatively small sample size. Accordingly, our findings should be taken as preliminary. Longitudinal study with a big sample size is needed in the future to achieve more convincing and accurate results. Second, MMSE and MoCA tests used in this study are 2 simple screening scales to identify MCI. More screening methods are needed in the future study. Third, we did not consider other possible influential factors on the brain in this study, such as the impact of medication and food intake. In further studies, we will include these factors.

## 5 Conclusion

In this study, we found that T2DM could influence the cerebral cortical thickness of several brain regions, including in the left posterior cingulate gyrus, parahippocampal gyrus, pars triangularis, right isthmus cingulate gyrus, middle temporal gyrus, paracentral lobule, transverse temporal gyrus, and pars opercularis. The cortical thickness reduction of the right pars opercularis may be a biomarker of cognitive impairment and play an important role in its pathophysiological mechanism.

## Author contributions

**Conceptualization:** Chang Li, Jian Wang.

**Data curation:** Chang Li, Qifang Yang, Bin Wang.

**Formal analysis:** Chang Li, Zhiwei Zuo.

**Writing – original draft:** Chang Li, Chuanming Li, Bin Wang, Xuntao Yin, Yuqi Lai.

**Funding acquisition:** Chuanming Li, Jian Wang.

**Methodology:** Chuanming Li, Qifang Yang, Xuntao Yin.

**Writing – review & editing:** Xuntao Yin, Jian Wang.

**Software:** Zhiwei Zuo, Xiaofei Hu.

**Project administration:** Jian Wang.
